# Real-World Dupilumab in Type 2 Chronic Obstructive Pulmonary Disease (COPD): A Single-Centre Compassionate-Use Case Series

**DOI:** 10.3390/biomedicines14071416

**Published:** 2026-06-23

**Authors:** Pier-Valerio Mari, Lorenzo Carriera, Alberto Ricci, Angelo Coppola, Simone Ielo, Alessandro D’Occhio, Armando Edoardo Ibello, Veronica Ojetti

**Affiliations:** 1 Internal Medicine, San Carlo di Nancy Hospital, Via Aurelia 275, 00165 Rome, Italy; vojetti@gvmnet.it; 2 Faculty of Medicine and Surgery, Università Cattolica del Sacro Cuore, 00168 Rome, Italy; lorenzocarriera@gmail.com; 3 Department of Pulmonology and Sub-Intensive Respiratory Unit, Santa Maria della Misericordia Hospital, 06156 Perugia, Italy; 4 Division of Pneumology, Department of Clinical and Molecular Medicine, Sapienza University of Rome, AOU Sant’Andrea, 00189 Rome, Italy; alberto.ricci@uniroma1.it; 5 UOC Pneumologia, Ospedale San Filippo Neri–ASL Roma 1, 00135 Rome, Italy; coppolangelo@gmail.com; 6 Faculty of Medicine, UniCamillus International Medical University of Rome, 00131 Rome, Italy; aledoc1999@gmail.com (A.D.); ibelloarmando@gmail.com (A.E.I.); 7 UOC Pneumologia e UTIP, Ospedale San Donato, USL Toscana SudEst, 52100 Arezzo, Italy; simone.ielo@uslsudest.toscana.it

**Keywords:** COPD, dupilumab, type 2 inflammation, eosinophils, chronic bronchitis, mucus hypersecretion, CAT, treatable traits, precision medicine

## Abstract

**Background**: Dupilumab, a monoclonal antibody blocking IL-4Rα, has recently demonstrated efficacy in patients with type 2 (T2)-inflamed chronic obstructive pulmonary disease (COPD) in the BOREAS and NOTUS trials. Real-world experience in older patients with predominant chronic bronchitis phenotype remains limited. **Methods**: We report a single-centre case series of 12 consecutive patients with T2-inflamed COPD treated with dupilumab 300 mg every two weeks under a compassionate-use programme at San Carlo di Nancy Hospital, Rome (first administration: April 2025). Eligibility required ≥2 moderate or ≥1 severe exacerbation in the prior 12 months despite triple inhaled therapy and a blood eosinophil count ≥300 cells/µL. Follow-up ranged from 3 to 12 months, with 6 months pre-specified as the primary analysis timepoint; data at 9 and 12 months are reported as descriptive observations. Endpoints included paired changes in annualised exacerbation rate (AER), CAT score and item-level CAT, and FEV_1_, with exploratory univariate Spearman analyses of candidate baseline predictors of response. **Results**: The cohort was elderly (mean age 73.6 ± 5.2 years, range 65–82), predominantly female (8/12, 67%) and characterised by a chronic bronchitis phenotype with high symptom burden (mean baseline CAT 22.8 ± 7.5; CAT item 2 [phlegm] median 3, IQR 3–4). Severe exacerbations decreased significantly (Wilcoxon *p* = 0.0156; mean AER 0.75 → 0.19 events/patient-year; 6/12 improved, 0/12 worsened). The mean cumulative function showed a standardised incidence ratio of 0.46 (95% CI 0.19–0.95; *p* = 0.033) versus the pre-dupilumab rate. Mean FEV_1_ increased by +66 mL at 1 month (*n* = 11, paired Wilcoxon *p* = 0.025), +78 mL at 3 months (*n* = 10, *p* = 0.082) and +120 mL at 6 months (*n* = 10, *p* = 0.007). Total CAT decreased from 22.9 to 12.5 at 6 months (Friedman *p* = 0.0007), with the largest absolute reductions in item 2 (phlegm; Δ = −2.6 at 6 months, *p* < 0.001) and item 3 (chest tightness; Δ = −2.5 at 6 months, *p* = 0.002). Higher baseline CAT was associated with greater reduction in severe AER (Spearman ρ = −0.79, *p* = 0.002). **Conclusions**: In this elderly real-world cohort with phlegm-driven T2 COPD, dupilumab was associated with a significant decrease in severe exacerbations, a clinically meaningful gain in lung function and a marked improvement in mucus-related symptoms. Further studies are warranted to confirm these findings and to clarify whether the reduction in severe exacerbations translates into a measurable mortality benefit.

## 1. Introduction

Chronic obstructive pulmonary disease (COPD) remains a leading cause of morbidity and mortality worldwide, and exacerbations are the principal drivers of disease progression, hospitalisation and death [1,2,3]. Approximately one in three patients with COPD harbours a type 2 (T2) inflammatory signature, defined by elevated blood eosinophils, increased FeNO, or both [4]. This subgroup has historically had limited targeted therapeutic options beyond inhaled corticosteroids and, more recently, the anti-IL-5 monoclonal antibody mepolizumab evaluated in the METREX–METREO programme [5].

Dupilumab, a fully human monoclonal antibody that blocks the shared IL-4 receptor alpha subunit and consequently inhibits both IL-4 and IL-13 signalling, has reframed the therapeutic landscape [6]. The pivotal BOREAS and NOTUS [7,8] phase 3 trials demonstrated, in patients with T2-inflamed COPD on triple inhaled therapy, an approximately 30% reduction in the annualised rate of moderate or severe exacerbations (rate ratio 0.70, 95% CI 0.58–0.86 in BOREAS; rate ratio 0.66, 95% CI 0.54–0.82 in NOTUS) together with significant gains in pre-bronchodilator FEV1 at week 12 (+83 mL and +76 mL, respectively). The European Medicines Agency was the first regulatory authority to approve dupilumab for COPD on 3 July 2024 [9], followed by the U.S. Food and Drug Administration on 27 September 2024 [10]. Notwithstanding the strength of the trial evidence, real-world experience with dupilumab in COPD remains limited, particularly in elderly patients with the predominant chronic bronchitis phenotype, in whom mucus hypersecretion is a defining and debilitating treatable trait [11]. Mucus plugging—visible on imaging and clinically measurable through the CAT phlegm item—has emerged as a robust prognostic marker linked to lung function decline, exacerbation frequency and mortality [12,13], and represents a biologically plausible target for IL-13 blockade given the central role of this cytokine in goblet-cell metaplasia and MUC5AC overexpression [14,15]. Whether dupilumab translates this mechanistic premise into measurable real-world benefit, especially in older and more comorbid patients than those typically enrolled in trials, is currently unknown [16].

The aim of the present study was to describe the 6-month clinical, functional and patient-reported outcomes (with descriptive observation up to 12 months) of the first cohort of patients with T2-inflamed COPD treated with dupilumab under a compassionate-use programme at our centre, and to explore baseline characteristics potentially associated with the magnitude of response.

## 2. Materials and Methods

### 2.1. Study Design and Setting

This is a retrospective single-centre case series describing all consecutive patients with T2-inflamed COPD who initiated dupilumab under a compassionate-use programme at the Pulmonology Service of San Carlo di Nancy Hospital (GVM Care & Research, Rome, Italy) from 1 April 2025 onwards. The programme reflected the indication subsequently approved in the European Union and was conducted within standard clinical practice; ethical approval and informed consent for off-label/compassionate use and data analysis were obtained according to institutional and national regulations. The study is reported according to the relevant guidance for case series reporting [17].

### 2.2. Patients

Eligibility criteria reflected the population of the BOREAS and NOTUS pivotal trials and required: (i) a confirmed diagnosis of COPD with post-bronchodilator FEV1/FVC < 0.70; (ii) blood eosinophil count ≥ 300 cells/µL; (iii) ≥2 moderate or ≥1 severe exacerbation in the previous 12 months despite optimised triple inhaled therapy (long-acting β2-agonist + long-acting muscarinic antagonist + inhaled corticosteroid); (iv) symptomatic disease (CAT ≥ 10 or mMRC ≥ 2); and (v) the chronic bronchitis phenotype, with predominant cough and sputum production. Patients with a current asthma diagnosis or alpha-1 antitrypsin deficiency were not included.

### 2.3. Treatment

Dupilumab was administered subcutaneously at the standard dose of 300 mg every two weeks. All patients continued their background triple inhaled therapy throughout follow-up. Oral corticosteroids were prescribed only for the treatment of acute exacerbations.

### 2.4. Outcomes and Assessments

The pre-specified primary endpoint was the change in the annualised rate of moderate or severe exacerbations between the 12 months preceding dupilumab initiation and the on-treatment follow-up period. Secondary endpoints included separate analyses of moderate and severe exacerbations, change in pre-bronchodilator FEV1, change in CAT total and item-level scores, and mMRC. Spirometry and CAT were performed at scheduled visits every 3 months (baseline, 3, 6, 9 and 12 months; with an additional 1-month FEV1 assessment), allowing a ±4-week window around each nominal timepoint. A moderate exacerbation was defined as an event requiring systemic corticosteroids and/or antibiotics; a severe exacerbation as an event requiring hospitalisation or emergency department care, in line with GOLD definitions [3].

Spirometry was performed in accordance with the 2019 American Thoracic Society and European Respiratory Society technical standards [18]; predicted values, percent-predicted and z-scores for FEV1, and FVC were derived using the Global Lung Function Initiative 2012 (GLI 2012) [19] reference equations. GOLD severity grade was assigned according to FEV1 percent predicted (grade 1 ≥80%; grade 2 50–79%; grade 3 30–49%; grade 4 <30%). Measurements were performed on a CareFusion VMAX Encore spirometer (Vyaire Medical, Mettawa, IL, USA).

### 2.5. Statistical Analysis

Continuous variables are reported as mean ± standard deviation or median (interquartile range), as appropriate; categorical variables as counts and percentages. Annualised exacerbation rates (AERs) for the post-treatment period were calculated as the observed number of events divided by the patient-specific follow-up duration, multiplied by twelve. Pre/post comparisons used the paired Wilcoxon signed-rank test. Within-patient trajectories of FEV1 and CAT were compared with the Friedman test across timepoints with at least *n* ≥ 4. The mean cumulative function (MCF) of recurrent exacerbations on dupilumab was compared with the expected number derived from the pre-treatment AER, and a standardised incidence ratio (SIR) with exact 95% confidence interval was computed under a Poisson assumption. Given the small sample (*n* = 12), exploratory associations between candidate baseline variables (age, sex, current smoking, weight, baseline blood eosinophil count, baseline FEV1, baseline CAT, baseline CAT item 2 [phlegm]) and the magnitude of change in AER were assessed by Spearman rank correlation. We deliberately abstained from fitting a multivariable model; with twelve patients and a small number of events, any multivariable regression would be hopelessly over-parameterised and the resulting estimates statistically meaningless. Univariate associations should accordingly be interpreted as hypothesis-generating. A two-sided *p*-value below 0.05 was considered statistically significant. Analyses were performed using Python (SciPy) and R.

## 3. Results

### 3.1. Cohort Characteristics

Twelve patients were treated with dupilumab between April 2025 and the analysis cut-off. Baseline demographic, clinical and functional characteristics are summarised in Table 1. The cohort was elderly (mean age 73.6 ± 5.2 years; range 65–82) and predominantly female (8/12, 67%). Seven patients were former smokers and five were current smokers. All patients met the eosinophil threshold (median 350 cells/µL, IQR 318–445; range 300–680). Lung function was severely impaired (mean FEV1 0.93 ± 0.30 L; mean FEV1/FVC 0.47 ± 0.12), with a high symptom burden (mean CAT 22.8 ± 7.5; mMRC ≥ 3 in 7/12). The chronic bronchitis phenotype was confirmed by the distribution of the CAT phlegm item, with 9/12 patients (75%) scoring ≥ 3 and 5/12 (42%) scoring ≥ 4 at baseline. In the year preceding dupilumab, the cohort had experienced a total of 26 exacerbations (17 moderate, 9 severe), corresponding to a pre-treatment AER of 2.17 events/patient-year. One patient died at month 9 (Pt 1), as detailed below. According to GLI 2012 reference equations, mean FEV1 was 41.2 ± 11.7% predicted with a mean z-score of −3.27 ± 0.57; the distribution by GOLD grade was 4 patients in grade 2 (moderate), 5 in grade 3 (severe) and 3 in grade 4 (very severe).

### 3.2. Exacerbations: The Primary Endpoint

**Figure 1:**
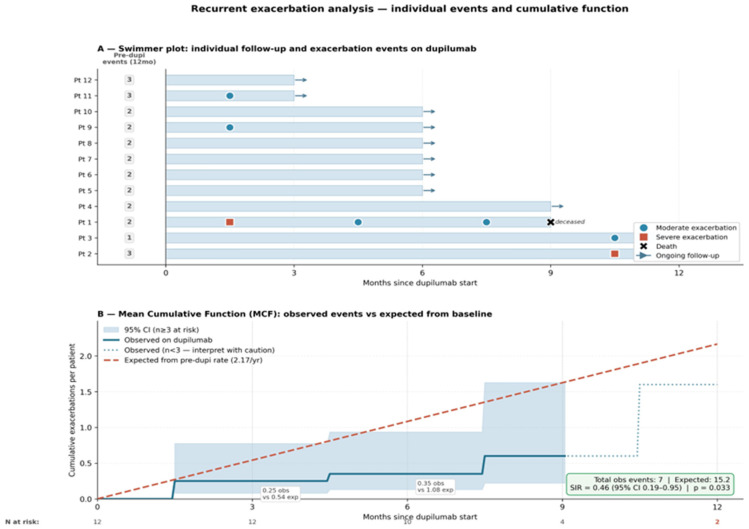
Recurrent-exacerbation analysis. (**A**) Swimmer plot showing individual follow-up duration and exacerbation events on dupilumab; numbers on the left indicate pre-treatment events in the prior 12 months; circles, moderate events; squares, severe events; arrows, ongoing follow-up; the cross indicates the death of Pt 1 at month 9. (**B**) Mean cumulative function (MCF) of exacerbations on dupilumab versus the expected curve derived from the pre-treatment annualised rate (2.17 events/patient-year); shaded area, 95% CI when ≥3 patients at risk; dotted line, period with <3 patients at risk (interpret with caution). The cumulative SIR over the entire follow-up was 0.46 (95% CI 0.19–0.95; *p* = 0.033).

**Figure 2:**
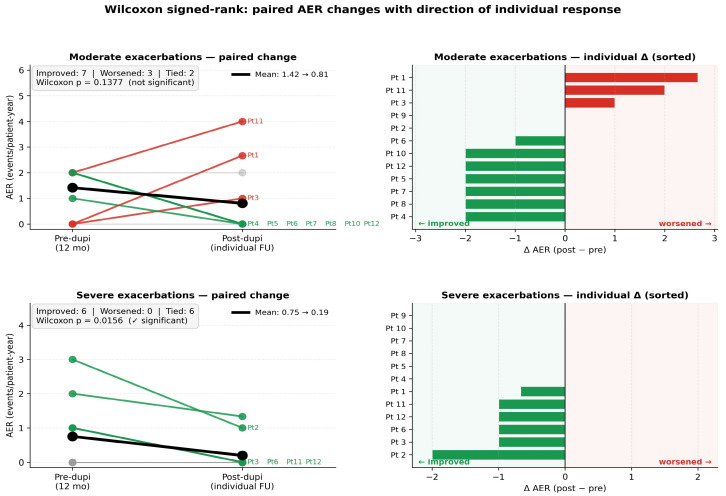
Paired changes in annualised exacerbation rate (AER) before and during dupilumab, separated by severity. Top row: moderate exacerbations (Wilcoxon *p* = 0.1377). Bottom row: severe exacerbations (Wilcoxon *p* = 0.0156). Left panels: individual paired trajectories with cohort mean (bold). Right panels: sorted individual Δ AER (post—pre); negative values indicate improvement. Pre-dupilumab AER was calculated over the 12 months before initiation; post-dupilumab AER was calculated over each patient’s individual follow-up period (range 3–12 months).

Patient-level event data, individual follow-up windows and the cumulative event function are shown in Figure 1. Total observed exacerbations on dupilumab were 7 against 15.2 expected from the pre-treatment AER, yielding an SIR of 0.46 (95% CI 0.19–0.95; *p* = 0.033). When stratified by severity, the picture was clearer (Figure 2). Moderate exacerbations decreased from a mean AER of 1.42 to 0.81 events/patient-year, with 7/12 patients improved, 3/12 worsened and 2/12 unchanged; the paired Wilcoxon test was not significant (*p* = 0.1377). Severe exacerbations, by contrast, decreased from 0.75 to 0.19 events/patient-year, with 6/12 improved, 0/12 worsened and 6/12 unchanged (most of these being patients with no severe events either before or during treatment); the difference reached statistical significance (Wilcoxon *p* = 0.0156). The single death occurred at month 9 in a 79-year-old woman (Pt 1) who experienced a severe exacerbation in the early treatment period and two further moderate events; she contributed disproportionately to the moderate-exacerbation arm and is the principal reason for the divergence between the moderate and severe trajectories.

### 3.3. Lung Function

**Figure 3:**
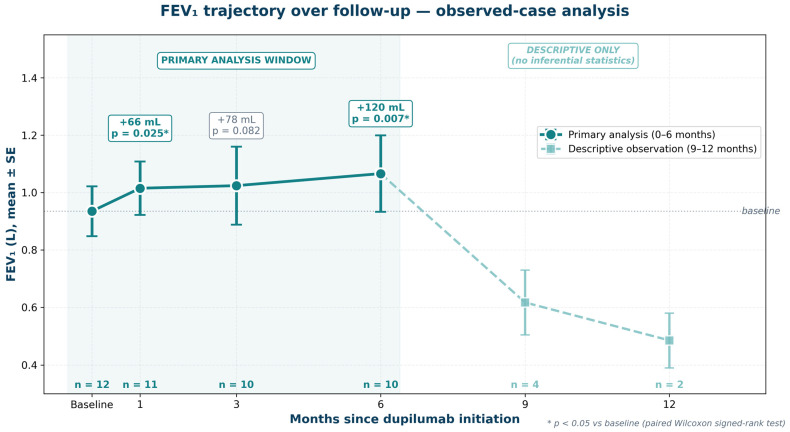
Mean absolute FEV1 trajectory under dupilumab. Group mean FEV1 ± SE; solid line, reliable data (*n* ≥ 10, baseline–6 months); dashed segment, sparse data (*n* = 4 at 9 months, *n* = 2 at 12 months). Annotations show mean Δ versus baseline at 1, 3 and 6 months; asterisks indicate *p* < 0.05 (paired Wilcoxon). The 3-month change (+78 mL) is consistent with the week-12 lung function gains reported in the BOREAS (+83 mL) and NOTUS (+65 mL) trials.

Mean FEV1 trajectory under dupilumab is shown in Figure 3. A statistically significant gain was observed already at month 1 (Δ = +66 mL, *p* = 0.025, *n* = 11), with further increase at month 3 (Δ = +78 mL, *p* = 0.082, *n* = 10) and a peak at month 6 (Δ = +120 mL, *p* = 0.007, *n* = 10). The 6-month gain is numerically larger than the +83 mL and +65 mL improvements reported at week 12 in BOREAS and NOTUS, and is plausible in a real-world cohort heavily skewed towards the chronic bronchitis phenotype, in which IL-13-driven mucus plugging and goblet-cell metaplasia constitute a particularly tractable mechanism of small-airway obstruction. Data at months 9 and 12 are limited (*n* = 4 and *n* = 2, respectively) and primarily reflect the trajectory of the most fragile patients still under follow-up; they should be interpreted with caution.

### 3.4. Symptom Burden: CAT Total and Item-Level Analysis

**Figure 4:**
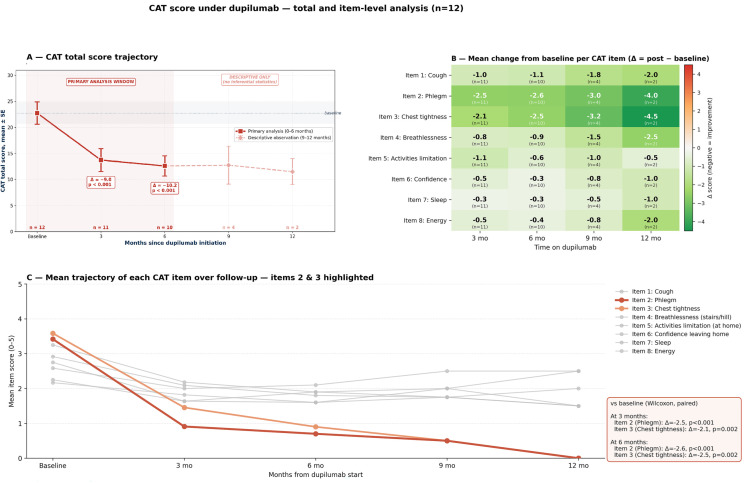
CAT response to dupilumab (*n* = 12). (**A**) Trajectory and mean ± SE (bold) of total CAT score. (**B**) Heatmap of mean Δ from baseline per CAT item; green, improvement; red, worsening; *n* shown in each cell. (**C**) Mean trajectory of each CAT item; items 2 (phlegm) and 3 (chest tightness) highlighted. The inset reports paired Wilcoxon results versus baseline at 3 and 6 months for the two highlighted items.

The total CAT score (Figure 4A) decreased rapidly from 22.8 ± 7.5 at baseline (*n* = 12) to 13.7 ± 7.3 at month 3 (*n* = 11) and 12.6 ± 6.2 at month 6 (*n* = 10); the within-patient change across the primary-analysis trajectory was statistically significant (Friedman *p* = 0.0007). CAT remained substantially below baseline at the late descriptive timepoints (mean 12.8, *n* = 4 at 9 months; mean 11.5, *n* = 2 at 12 months), although these latter estimates are based on too few patients to support formal inference. The mean change at month 6 (Δ = −10.2 points) is approximately five times the established minimal clinically important difference (MCID) of 2 points [20,21], and was reached at month 3 in the great majority of patients.

The item-level analysis (Figure 4B,C) provides the mechanistic correlate of this overall benefit. The two CAT items showing the largest and earliest improvement were item 2 (phlegm) and item 3 (chest tightness): item 2 decreased by Δ = −2.5 at month 3 and Δ = −2.6 at month 6 (paired Wilcoxon *p* < 0.001 at both timepoints), while item 3 decreased by Δ = −2.1 at month 3 (*p* = 0.002) and Δ = −2.5 at month 6 (*p* = 0.002). Improvements in cough (item 1), breathlessness (item 4) and energy (item 8) were of smaller magnitude and emerged later, while items 5–7 (activities, confidence, sleep) were the least responsive. This pattern is biologically coherent with selective blockade of IL-4/IL-13 signalling and pinpoints mucus and chest tightness as the mechanistic core of the symptomatic response.

### 3.5. Exploratory Predictors of Response

Table 2 reports univariate Spearman correlations between candidate baseline variables and the magnitude of change in AER (any, moderate and severe) and in 6-month FEV1. Three signals are noteworthy. First, baseline CAT correlated negatively with the change in severe AER (ρ = −0.79, *p* = 0.002): patients with the highest baseline symptom burden experienced the greatest reduction in severe events. Second, baseline FEV1 correlated positively with the change in severe AER (ρ = +0.64, *p* = 0.025): patients with worse baseline lung function had the largest reduction in severe events. Third, a non-significant trend was observed for higher baseline blood eosinophils to be associated with greater reduction in any-severity AER (ρ = −0.49, *p* = 0.107). Age, sex, weight, and the baseline phlegm item showed no significant associations with any of the outcomes; current smoking showed a borderline positive correlation with the change in moderate AER (ρ = +0.58, *p* = 0.050), suggesting that active smokers retained a residual moderate-event burden under treatment, although this did not extend to severe events. None of the candidate baseline variables predicted the 6-month FEV1 gain. These findings are exploratory and hypothesis-generating, given the small sample and the inherent multiplicity of testing.

## 4. Discussion

This real-world case series describes the first patients with T2-inflamed COPD treated with dupilumab at our centre under a compassionate-use programme. Three findings warrant emphasis: a statistically significant reduction in severe exacerbations, a clinically meaningful gain in lung function that exceeded the magnitude reported in BOREAS and NOTUS, and a marked, mechanistically plausible improvement in mucus-related symptoms.

The cohort is older and more functionally impaired than the trial populations: mean age 73.6 versus approximately 65 years in BOREAS, mean baseline FEV1 0.93 L versus approximately 1.5 L, and mean baseline CAT 22.8 versus approximately 19. Despite this, the response to dupilumab was preserved and, in some respects, more pronounced than expected. The 6-month gain in FEV1 of +120 mL is approximately fifty percent larger than the +83 mL observed at week 12 in BOREAS and the +76 mL observed in NOTUS. We hypothesise that the chronic bronchitis phenotype that dominates our cohort, captured by the very high baseline CAT phlegm item (median 3, IQR 3–4), provides a particularly large biological substrate for IL-4/IL-13 blockade; in patients whose airway obstruction is heavily driven by goblet-cell metaplasia and mucus plugging, removing the main driver of small-airway occlusion can produce a functional benefit beyond the average trial effect [22]. The item-level CAT analysis is consistent with this interpretation; items 2 (phlegm) and 3 (chest tightness) showed the largest and earliest decrease, while items more dependent on cardiopulmonary deconditioning and frailty (5–7) responded less.

The clinical significance of the observed reduction in severe exacerbations deserves a careful comment. While the change in moderate exacerbations did not reach statistical significance, the reduction in severe events was significant on the paired Wilcoxon test (*p* = 0.0156), with no patient worsening, and the SIR computed on the recurrent-event analysis was 0.46 (95% CI 0.19–0.95; *p* = 0.033). Severe exacerbations, defined here as events requiring hospitalisation or emergency-department care, are not a generic symptomatic outcome; they are the principal driver of mortality in COPD, with each severe event independently increasing the risk of death in the following months and years. A single severe exacerbation roughly doubles the short-term mortality risk, and patients who experience repeated severe events have a substantially higher long-term mortality than the general COPD population [4]. If dupilumab consistently reduces the rate of severe events in real-world patients with the T2 phenotype, as our data and the trial evidence converge to suggest, the implication is that the benefit could extend beyond symptom control to a measurable impact on survival. The single death observed in our cohort, in a 79-year-old patient with the lowest baseline FEV1 and a severe exacerbation occurring early in the treatment course, illustrates the residual vulnerability of these patients and underscores the need for adequately powered survival analyses in larger real-world cohorts.

The exploratory predictor analysis (Table 2) yielded two coherent signals. The positive correlation between baseline CAT and reduction in severe AER, together with the positive correlation between baseline FEV1 and Δ AER severe (i.e., patients with worse lung function showed greater absolute reduction in severe events), can be parsimoniously interpreted as a regression-toward-the-mean component combined with a genuine biological signal; the patients with the highest disease activity at baseline have the largest absolute room for improvement, and dupilumab appears to capture that room when the underlying inflammatory mechanism is T2 in nature. The trend towards greater benefit in patients with higher baseline eosinophils, although not statistically significant, is consistent with the dose-response observed in the trial programme. We deliberately did not fit a multivariable model; with twelve patients and a small number of events, any such model would be hopelessly over-parameterised and would generate misleadingly precise estimates. In the spirit of the treatable-traits framework [23], these univariate signals should be read as hypotheses for prospective registry studies, not as effect estimates.

Our findings sit comfortably within the broader effort to refine T2 phenotyping in COPD beyond blood eosinophils alone. Mucus plugging on imaging is a robust prognostic biomarker in heavy-smoker cohorts and correlates with FEV1 decline, exacerbation frequency and mortality. The CAT phlegm item, despite its inherent simplicity, captures a closely related construct in routine clinical practice and may prove a pragmatic candidate for treatable-trait identification. The fact that, in our cohort, the same item showed the largest and earliest improvement under dupilumab provides preliminary evidence that the phlegm-driven phenotype is not only a prognostic marker but also a predictive one. Whether this pattern generalises to larger real-world populations and translates into a survival benefit is, in our view, the most important question to be addressed by future studies.

### Study Limitations

Several limitations should be acknowledged. First, the sample size is small (*n* = 12) and the follow-up is necessarily uneven, with only two patients reaching 12 months at the time of analysis. This constrains both the precision of the estimates and the reliability of the late timepoints; accordingly, we have limited inferential analyses to the 6-month primary timepoint, while data at 9 and 12 months are presented as descriptive observations only. Estimates beyond 6 months should be interpreted as preliminary signals worthy of confirmation in larger cohorts and longer follow-up, especially for FEV1 and total CAT. Second, the design is single-arm and retrospective, with no concurrent control; the comparison with the patient’s own pre-treatment year is the only available counterfactual, and regression-to-the-mean cannot be excluded as a partial contributor to the observed improvement, particularly in the most symptomatic patients. Third, the cohort is older and more frail than typical trial populations, which strengthens the external validity in the elderly real-world setting but may limit generalisability to younger patients. Fourth, FeNO and 6-min walk test were available in only six patients, precluding formal subgroup analyses on these variables. Fifth, the exploratory predictor analyses are univariate and rest on a small sample: they are intended to generate hypotheses for prospective registry studies and not to support causal claims.

## 5. Conclusions

In this elderly real-world cohort with T2-inflamed COPD and a predominant chronic bronchitis phenotype, dupilumab was associated with a statistically significant reduction in severe exacerbations, a clinically meaningful gain in lung function that compares favourably with the pivotal trial evidence, and a marked, mechanistically plausible improvement in mucus-related symptoms. The pattern of response—largest in patients with highest baseline symptom burden and lowest lung function—supports a precision-medicine model in which CAT phlegm-driven phenotyping complements eosinophil-based selection. Further studies are warranted to confirm these findings in larger registries, to clarify whether the observed reduction in severe exacerbations translates into a measurable mortality benefit, and to position dupilumab within the broader landscape of biologic therapy for COPD.

## Figures and Tables

**Table 1:** Baseline demographic, clinical and functional characteristics of the cohort (*n* = 12).

Characteristic	Value (*n* = 12)
Age, years—mean ± SD (range)	73.6 ± 5.2 (65–82)
Female sex—*n* (%)	8 (66.7)
Body weight, kg—mean ± SD	69.0 ± 15.5
Height, cm—mean ± SD (range)	163.0 ± 9.7 (150–182)
Smoking status—*n*	Current 5/Former 7
Blood eosinophils, cells/µL—median (IQR); range	350 (318–445); 300–680
FeNO, ppb—median (range)	26.5 (8–100)
FEV_1_, L—mean ± SD (range)	0.93 ± 0.30 (0.49–1.31)
FEV_1_% predicted (GLI 2012)—mean ± SD	41.2 ± 11.7
FEV_1_ z-score (GLI 2012)—mean ± SD	−3.27 ± 0.57
GOLD grade—*n* (%)	G2: 4 (33.3); G3: 5 (41.7); G4: 3 (25.0)
FVC, L—mean ± SD	2.04 ± 0.63
FEV_1_/FVC—mean ± SD	0.47 ± 0.12
6-min walk test, metres—mean (range)	343 (275–420)
mMRC dyspnoea—distribution (2/3/4)	5/3/4
CAT total—mean ± SD; median (range)	22.8 ± 7.5; 22 (12–37)
CAT item 2 (phlegm)—median (IQR); range	3 (3–4); 2–5
CAT item 2 ≥ 3—*n* (%)	9 (75.0)
CAT item 2 ≥ 4—*n* (%)	5 (41.7)
Pre-treatment moderate exacerbations/12 mo—mean; total	1.42; 17
Pre-treatment severe exacerbations/12 mo—mean; total	0.75; 9
Pre-treatment AER (any)—events/patient-year	2.17
Maintenance OCS at baseline—*n* (%)	1 (8.3)

Abbreviations: AER, annualised exacerbation rate; CAT, COPD Assessment Test; FeNO, fractional exhaled nitric oxide; FEV_1_, forced expiratory volume in 1 s; FVC, forced vital capacity; IQR, interquartile range; mMRC, modified Medical Research Council; OCS, oral corticosteroids; SD, standard deviation.

**Table 2:** Univariate Spearman correlations between baseline variables and changes in exacerbation rate and lung function. Negative ρ for AER endpoints indicates that higher predictor values are associated with greater reduction (improvement). Statistically significant correlations (*p* < 0.05) are marked in bold.

Predictor	Outcome	ρ	*p*	*n*
Age (years)	Δ AER any	+0.17	0.609	12
Sex (female = 1)	Δ AER any	+0.32	0.319	12
Current smoking (yes = 1)	Δ AER any	+0.55	0.066	12
Baseline EOS (cells/µL)	Δ AER any	−0.49	0.107	12
	Δ AER moderate	−0.32	0.309	12
	Δ AER severe	−0.11	0.736	12
Baseline FEV_1_ (L)	Δ AER any	−0.17	0.592	12
	Δ AER moderate	−0.48	0.116	12
	Δ AER severe	+0.64	**0.025**	12
Baseline CAT	Δ AER any	+0.34	0.285	12
	Δ AER moderate	+0.61	**0.034**	12
	Δ AER severe	−0.79	**0.002**	12

Abbreviations: AER, annualised exacerbation rate; CAT, COPD Assessment Test; EOS, blood eosinophils; FEV_1_, forced expiratory volume in 1 s; ρ, Spearman rank correlation coefficient.

## Data Availability

The raw data supporting the conclusions of this article will be made available by the authors on request.
